# Dental surgery for patients with bleeding disorder of unknown cause

**DOI:** 10.1038/s41415-025-8815-z

**Published:** 2025-09-26

**Authors:** Ishfaq Khan

**Affiliations:** Honorary Assistant Professor, College of Medicine and Health, University of Birmingham, Dentistry, Birmingham, UK; Senior Dental Surgeon (Special Care Dentistry), Midlands Partnership University NHS Foundation Trust, Dental Services, Cannock Hospital Dental Department, Staffordshire, United Kingdom

## Abstract

Bleeding disorder of unknown cause (BDUC) is a haematological diagnosis characterised by excessive bleeding in patients without a known aetiology. Patients with BDUC exhibit a bleeding phenotype like other mild bleeding disorders, such as haemophilia or von Willebrand disease, and may experience prolonged bleeding following dental surgery. Treatment options for patients requiring dental surgery include tranexamic acid, desmopressin, or a combination of both. For more invasive surgeries, fresh frozen plasma, platelet transfusions, or recombinant factor VIIa may be necessary. Managing patients with BDUC requires a multidisciplinary approach and the involvement of haematology to prevent bleeding complications following dental surgery.

**Supplementary Information:**

Zusatzmaterial online: Zu diesem Beitrag sind unter 10.1038/s41415-025-8815-z für autorisierte Leser zusätzliche Dateien abrufbar.

## Key points

- Presents a summary of bleeding disorder of unknown cause and its significance for dentists.
- Discusses potential oral signs of bleeding disorder of unknown cause and the risk of bleeding following dental surgery.
- Analyses current studies and outlines the current recommended management of patients with bleeding disorder of unknown cause who require dental surgery.

**Supplementary Information:**

Zusatzmaterial online: Zu diesem Beitrag sind unter 10.1038/s41415-025-8815-z für autorisierte Leser zusätzliche Dateien abrufbar.

## Introduction

Patients with mild bleeding disorders (MBDs) represent a significant patient population for dentists. Common patient groups include those with von Willebrand disease, haemophilia, or platelet defects. Additionally, patients taking medications, such as vitamin K antagonists, direct oral anticoagulants, or antiplatelets, may experience excessive bleeding following dental surgery. These patient groups have established and recognised dental management guidelines.^1^^,^^2^

However, up to 60% of patients with an MBD may be diagnosed with a bleeding disorder of unknown cause (BDUC),^3^ where there is no identifiable cause following haematological evaluation (all current known tests, such as full blood count, prothrombin time, activated partial thromboplastin time, factor VIII, or von Willebrand factor [vWF] antigen, are negative). BDUC was previously termed ‘unclassified bleeding disorder' and ‘bleeding of unknown cause'.

BDUC is a diagnosis of exclusion and may be defined as abnormal or excessive bleeding in a patient presenting with normal haemostatic evaluation and where no known cause can be identified.^4^ This may complicate dental management as patients may present with a medical history not suggestive of a bleeding tendency to their dentist and experience bleeding before a diagnosis of BDUC has been made. The incidence of bleeding in patients with BDUC following dental surgery can range from 36-84%.^5^

The aim of this paper is to increase awareness of BDUC among dentists, analyse current published studies and guidelines, and outline current recommendations for the management of patients requiring dental surgery.

## BDUC clinical presentation

BDUC has a similar bleeding phenotype to other MBDs and may present as epistaxis, haematomas, haemarthrosis, menorrhagia, postpartum menorrhagia, haematuria and post-surgical bleeding.^6^ It is more common in women.^4^

There is evidence that patients with BDUC may suffer from reduced health-related quality of life, such as role limitation, reduced mental health, impaired social function and bodily pain.^7^ They may experience similar barriers to dentistry as other patients with MBDs (e.g., haemophilia), including reduced access due to physical impairment, avoiding dental treatment due to the fear of bleeding, and dentists unwilling to treat them due to a lack of awareness of their condition.

## Diagnosis of BDUC

Diagnosis of BDUC involves a full medical history (including medications), physical exam, family history, surgical history (including any complications) and haemostatic evaluation, which should include: a full blood count; prothrombin time; activated partial thromboplastin time; thrombin time; vWF antigen and function; coagulation factors VIII, IX and XI; and platelet light transmission aggregometry (LTA) haemostasis testing.^6^ If LTA is normal, a validated platelet secretion assay may be considered. If this is also normal, BDUC may be diagnosed.^6^ More advanced tests may also be employed to rule out other rare causes of bleeding.^8^

The pathophysiology of BDUC remains unknown. It is most likely due to a heterogenous group of different underlying defects (e.g., low vWF, coagulation factor deficiencies, hyperfibrinolysis etc.) causing bleeding symptoms, or a collection of disorders that are yet to be identified.^4^^,^^5^ Up to 40% of BDUC patients have a family history of bleeding.^5^

Differential diagnoses of BDUC include mild haemophilia, von Willebrand disease, platelet function disorders, connective tissue or vascular disorders (e.g., Ehlers-Danlos syndrome [EDS] or hereditary haemorrhagic telangiectasia) and ascorbic acid deficiency.^4^

Dentists may be able to aid in diagnosis. For example, patients with a clear or non-suggestive medical history for bleeding who bleed excessively following dental extractions - unresponsive to local measures and possibly requiring hospitalisation - should be referred to haematology for assessment for a potential diagnosis of BDUC.

## Oral signs of BDUC

Oral signs of BDUC may include significant bleeding from dental extractions,^9^^,^^10^ and a history of previous bleeding following dental extractions may be predictive of future bleeding from dental treatment.^11^ Other oral signs include oral mucosal bleeding (ranging from 19-53%),^5^ bruising/petechiae of the oral mucosa and gingival bleeding in the absence of periodontal disease (including during toothbrushing).^12^ This may contribute to neglect of oral health, leading to dental disease, which can necessitate invasive dental surgery.

BDUC can be associated with anaemia and iron deficiency,^6^ which may translate to angular cheilitis, glossodynia, mucosal pallor, ulceration and glossitis intra-orally. A recent study suggested patients with BDUC may have a higher incidence of ascorbic acid deficiency and hypermobility (potentially consistent with a diagnosis of hypermobile EDS).^13^ Oral signs associated with ascorbic acid deficiency include gingival manifestations, such as bleeding, swelling and hypertrophy, as well as loosening or loss of teeth. Hypermobile EDS may present with oral ulceration, temporomandibular joint dislocation, microdontia, enamel and dentine defects, pulp calcifications and root abnormalities.

## Assessing bleeding risk before invasive dental surgery

Assessing bleeding risk before invasive dental surgery involves evaluating the patient's medical, surgical and pharmacological history, the need for specialist haematological treatments, the complexity and invasiveness of the procedure, and the dentist's ability to safely carry out the treatment.

Key considerations in the patient's history include any previous episodes of prolonged bleeding following trauma, surgery, or dental procedures, as well as a family history of coagulation disorders. Certain medications, such as antiplatelets, anticoagulants, selective serotonin reuptake inhibitors and non-steroidal anti-inflammatory drugs, can increase bleeding risk. Furthermore, underlying medical conditions, like liver disease, advanced heart failure and alcoholism are important risk factors.

Operator and procedural risks are also important. Evidence suggests that experienced practitioners, familiar with the treatment required or specialists, are less likely to encounter complications.^14^ A useful tool for assessing bleeding risk (not associated with medications) and the need for referral in patients before invasive dental surgery, includes the HEMSTOP (Haematoma, haEmorrhage, Menorrhagia, Surgery, Tooth extraction, Obstetrics, and Parents) questionnaire (Table 1).^15^ A HEMSTOP score of 2 or higher suggests an increased risk of perioperative bleeding. Such cases require further investigation or management, which may involve consulting a haematologist and referral to a hospital setting.^15^

**Table 1:** HEMSTOP (Haematoma, haEmorrhage, Menorrhagia, Surgery, Tooth extraction, Obstetrics, and Parents) questionnaire to assess bleeding risk^15^

**Question**	**Applies to**	**Point for ‘yes'**
Have you ever consulted a doctor or received treatment for prolonged or unusual bleeding (such as nosebleeds, minor wounds)?	Male/female	1
Do you experience bruises or haematomas larger than 2 cm without trauma or severe bruising after minor trauma?	Male/female	1
After a tooth extraction, have you ever experienced prolonged bleeding requiring medical or dental consultation?	Male/female	1
Have you experienced excessive bleeding during or after surgery?	Male/female	1
Is there anyone in your family who suffers from a coagulation disease (e.g., BDUC, haemophilia, von Willebrand disease)?	Male/female	1
Have you ever consulted a doctor or received treatment for heavy or prolonged menstrual periods (e.g., contraceptive pill, iron supplements)?	Female	1
Did you experience prolonged or excessive bleeding after delivery?	Female	1
Interpretation: <2 points = low risk of bleeding, no further investigations needed; ≥2 points = increased risk of bleeding, further investigations recommended. Maximum score: 7 for female; 5 for male only. This table uses ‘female' to reflect the language used in the original study, while recognising that not all people who experience menstrual periods or can get pregnant identify as female, and not all those who identify as female experience menstrual periods or can get pregnant.		

## Current recommendations and review of the literature of BDUC

The 2024 guidelines from the International Society on Thrombosis and Haemostasis (ISTH)^6^ provide only general recommendations on how to manage BDUC patients requiring surgery.^16^ Treatment options for BDUC patients include observation only, tranexamic acid (TXA) only, TXA and desmopressin (DDAVP), and for more invasive surgery, fresh frozen plasma, platelet transfusions, or recombinant factor VIIa may be considered.^6^

The literature was reviewed and five published studies were identified that looked at patients with BDUC requiring invasive procedures.^9^^,^^17^^,^^18^^,^^19^^,^^20^ Considering these studies, how patients with BDUC were managed for dental/maxillofacial surgery were specifically analysed (see online Supplementary Information and online Supplementary Table 1).

## Observations

All patients requiring dental/maxillofacial surgery were treated with either TXA only, DDAVP only, or a combination of TXA and DDAVP, or in some cases, no haemostatic prophylaxis was provided. No dental/maxillofacial patient with BDUC was treated with fresh frozen plasma, platelet transfusions or recombinant factor VIIa cover.

Of the patients who received no haemostatic prophylaxis for dental extractions, complications included bleeding two days post-extraction and infected haematoma.^9^ In another study, a patient suffered minor bleeding following dental extraction, but in the same study, a patient who had received no cover reported no bleeding events post-dental extraction.^17^

Of the patients who received TXA only, two patients required additional DDAVP due to bleeding following dental extraction.^9^^,^^18^ One patient developed severe bleeding after a dental extraction^18^ and another patient developed major bleeding following jaw osteotomy.^17^ There was also a case of a patient who suffered an allergic reaction to TXA and later received DDAVP.^9^

The patients treated with DDAVP only or a combination of TXA and DDAVP suffered no bleeding events; however, one patient developed hyponatraemia and presyncope attributed to DDAVP.^19^

## Discussion

From the published studies, the general conclusions for dental surgery that can be made are that simple extractions may be covered by TXA only; however, surgical or multiple extractions may require TXA and DDAVP or DDAVP only. One patient who received a gingival graft was treated with no bleeding complications but specific details on the haemostatic prophylaxis (most patients in the study were treated with TXA or aminocaproic acid) are not reported.^20^

Only one maxillofacial surgical procedure was reported in the published studies (jaw osteotomy), which resulted in major bleeding after being covered by TXA only.^17^ Maxillofacial patients may be more likely to require fresh frozen plasma, platelet transfusions, or recombinant factor VIIa following consultation with haematology.

**Fig. 1:**
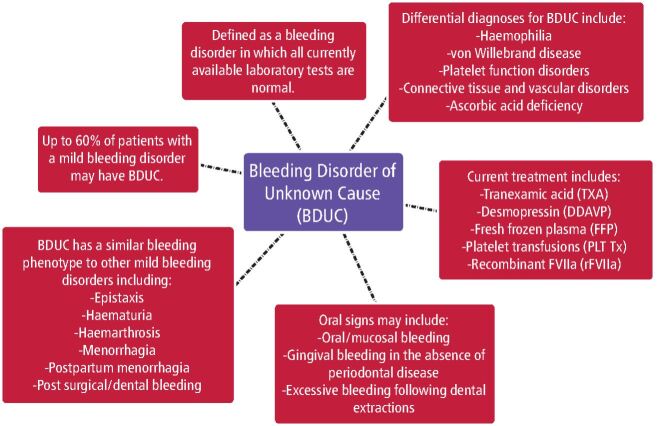
A summary of BDUC for dentists^3^^,^^4^^,^^5^

It is important to emphasise to BDUC patients that spontaneous life-threatening bleeding from BDUC is very rare^18^ and that they can have dental surgery when indicated with appropriate precautions. Patients with BDUC who require dental extractions or other invasive dental procedures should be evaluated by a haematologist before treatment. These patients may need TXA or DDAVP, which are not readily available in general or community dental settings, and may require referral to a hospital setting (see Figure 1 for a summary of BDUC).^3^^,^^4^^,^^5^

Hospitals offer access to advanced diagnostic tools, haemostatic agents, multidisciplinary teams and immediate emergency interventions, ensuring comprehensive evaluation and effective management of bleeding risks. Additionally, the expertise of haematologists, who predominantly work in hospital settings, plays a critical role in optimising treatment strategies and creating tailored haemostatic plans, particularly given the uncertain pathophysiology of BDUC and its unpredictable bleeding patterns. Consideration should also be made to refer patients to dental specialists like special care dentists.

Local measures and provisions used for other MBDs can be applied to BDUC. This includes: patient education on how their condition affects dental care; intensive prevention and earlier dental recall to avoid the need for invasive dental surgery; considering less invasive options for dental care (such as root canal therapy); atraumatic surgical technique; suturing; haemostatic dressings; and avoiding analgesia that may promote bleeding.^1^ Other considerations include sedation options, for example, bleeding from cannulation (bleeding risk may be mitigated by ultrasound guidance)^14^^,^^21^ or very rarely, epistaxis associated with inhalational sedation.^22^

It may be wise to treat BDUC patients early in the day and the working week so that any complications can be treated within normal working hours and review the patient with a tele-video consultation later in the day to ensure no complications post-dental surgery.

Haemostatic management of BDUC requires careful assessment. In the published studies, adverse outcomes included an allergic reaction to TXA (etamsylate [diethylamine 2,5-dihydroxy-benzenesulfonate] may be used as an alternative),^9^ and in one study, DDAVP was associated with hyponatraemia and presyncope.^19^ TXA may also be associated with visual impairment and seizures, while fresh frozen plasma can lead to skin reactions, transfusion-related infections, and injuries. Platelets may result in alloimmunisation, and recombinant factor VIIa can cause allergic reactions, embolism and thrombosis.^23^ Emerging treatments for BDUC include concizumab, which is administered subcutaneously and inhibits tissue factor pathway inhibitor, enhancing thrombin generation and potentially offering a better safety profile compared to other management options.^24^

### Limitations

The definition of BDUC was only recently standardised by the ISTH, meaning earlier studies on the condition may not fully comply with this definition. Additionally, as previously stated, BDUC has been referred to by several names, including ‘unclassified bleeding disorder' and ‘bleeding of unknown cause'. Such variations in terminology have likely influenced the consistency and progress of research into BDUC, potentially complicating the comparison and interpretation of findings across studies.^6^ The new ISTH clinical guidelines aim to effectuate a standardised approach to diagnosing and managing BDUC.

The current evidence base is limited to retrospective studies and the reported limitations of each of the studies are displayed in online Supplementary Table 1. Other limitations to consider include that the studies were not completed by dentists, and certain information is not clear or absent in some studies, including: i) the experience and specialty of the dentist completing the treatment; ii) the settings in which treatments were completed; iii) if any local measures were used pre- and post-operatively (i.e., haemostatic dressings or sutures); iv) the complexity of the extractions (e.g., complicating factors such as hypercementosis); and v) the full medical history of the patient. No studies have examined the risk of bleeding associated with other types of dental-surgical interventions such as restorations, professional mechanical plaque removal or periodontal surgery, block anaesthesia, and root canal therapy.

## Conclusion

Close collaboration with haematology is imperative for the management of patients with BDUC undergoing dental surgery. TXA appears to be a safe haemostatic agent for simple dental extractions; however, more invasive procedures may necessitate additional haemostatic support with desmopressin. Currently, there is insufficient evidence to endorse the routine use of fresh frozen plasma, platelet transfusions, or recombinant factor VIIa for dental surgery in BDUC patients; although, this may differ for maxillofacial surgeries. It is critical for dentists to recognise BDUC and its potential impact on dental surgery. As the body of knowledge and research on BDUC expands, there is a pressing need for prospective studies by dentists to clarify the haemostatic requirements of BDUC patients undergoing dental interventions.

## Supplementary Information

Supplementary Information (PDF 137KB)

Supplementary Table 1 (PDF 119KB)
