# Supplementary material for: Dental surgery for patients with bleeding disorder of unknown cause
Source: Br Dent J. 2025 Sep 26;239(6):402–5. doi: 10.1038/s41415-025-8815-z (PMC12474535; doi:10.1038/s41415-025-8815-z)
Supplement: Supplementary file 1 — Supplementary Information (PDF 137KB) [file 41415_2025_8815_MOESM1_ESM.pdf]

https://doi.org/10.1038/s41415-025-8815-z

SUPPLEMENTARY INFORMATION

The supplementary information that follows was gathered from two publications that offered additional detail on the patient's characteristics and the dental or maxillofacial procedures that were performed.

Obaji et al, 2016[9]:

Age/gender	Family history of bleeding	No of dental extractions/other treatment performed	Haemostatic prophylaxis	Complications
44/F	No	1	TXA	No bleeding
24/F	Yes	1	Desmo	Allergy to TXA, therefore dental extraction completed with desmopressin. The patient later had subsequent vaginal delivery with etamsylate and desmopressin.
72/F	Yes	3	TXA	No bleeding
33/F	Yes	2	TXA	No bleeding
49/F	No	1	TXA	No bleeding
52/F	Yes	1	No cover	Bleeding 2 days post extraction, infected haematoma (NOTE family history of bleeding issues)
50/F	No	1	TXA	No bleeding
54/F	No	1	TXA	No bleeding
83/M	Yes	Trigeminal nerve injection	TXA	No bleeding
59/F	Yes	3	2 (TXA)<br>1(TXA + Desmo)	Bleeding with TXA alone, Desmopressin added thereafter for third extraction (no bleeding)
20/F	Yes	1	Desmo + TXA	No bleeding
42/F	Unknown	1	Desmo + TXA	No bleeding
40/F	No	1	TXA	No bleeding
53/F	Yes	2	TXA	No bleeding

TXA= Tranexamic acid, Desmo= Desmopressin

Dental and Maxillofacial Treatment Summary

Dental Patients:
A total of **13 patients** underwent dental extractions, with the following haemostatic management:

- **No haemostatic cover**: 1 patient
- **Tranexamic acid (TXA) only**: 8 patients
- **DDAVP (Desmopressin) only**: 1 patient
- **Combination of TXA and DDAVP**: 3 patients

Maxillofacial Treatment:

- **Trigeminal nerve injection** was administered to **one patient**. *Note: The clinical indication for this procedure was not disclosed.*

https://doi.org/10.1038/s41415-025-8815-z

<u>Veen et al, 2021[17]:</u>

Definitions and classification of low bleeding risk surgical procedures (according to study):

Low bleeding risk surgical procedures	Procedures of the eyes, skin, nose, ears, and distal extremities as well as those pertaining to **dental**, perineal, and inguinal areas (e.g. inguinal hernia repair, myringotomy, and dilatation and curettage).

Outcome of BDUC patients receiving dental/maxillofacial surgery prophylactically treated *with tranexamic acid (TXA) and/or DDAVP (Desmopressin)*:

Age/Gender	Procedure	Classified risk by study	Treatment	Outcome
36/F	Tooth extraction	Low	Desmopressin	No bleeding
21/F	Tooth extraction	Low	Desmopressin and TXA	No bleeding
40/M	Tooth extraction	Low	TXA	No bleeding
72/M	Tooth extraction	Low	TXA	No bleeding
30/M	Extensive osteotomy of the jaw	Low	TXA	Major bleeding

DDAVP (desmopressin) = 1-deamino-8-D-arginine vasopressin, TXA= Tranexamic acid.

Outcome of BDUC patients receiving dental/maxillofacial surgery receiving *no prophylactic treatment*:

Age/Gender	Procedure	Classified risk by study	Treatment	Outcome
80/F	Tooth extraction	Low	No treatment	No bleeding
40/M	Tooth extraction	Low	No treatment	Clinically relevant minor bleeding

<u>Dental and Maxillofacial Treatment Summary</u>

Dental Patients:
A total of **6 patients** underwent dental extractions, with the following haemostatic management:

- **No haemostatic cover**: 2 patients
- **Tranexamic acid (TXA) only**: 2 patients
- **DDAVP (Desmopressin) only**: 1 patient
- **Combination of TXA and DDAVP**: 1 patient

Maxillofacial Treatment:

- **Jaw osteotomy** was performed on **one patient**.
