# Supplementary material for: Dental surgery for patients with bleeding disorder of unknown cause
Source: Br Dent J. 2025 Sep 26;239(6):402–5. doi: 10.1038/s41415-025-8815-z (PMC12474535; doi:10.1038/s41415-025-8815-z)
Supplement: Supplementary file 2 — Supplementary Table 1 (PDF 119KB) [file 41415_2025_8815_MOESM2_ESM.pdf]

https://doi.org/10.1038/s41415-025-8815-z

SI Table 1- The management of patients with BDUC who required dental/maxillofacial surgery from published studies.

Study	Type of study	Definition of BDUC used in study	Number of patients/procedures	Haemostatic challenge (dental/maxillofacial)	Haemostatic prophylaxis	Bleeding events/complications associated with haemostatic prophylaxis treatment	Limitations reported in study	Conclusions from study
Obaji et al, 2016[9]	Retrospective cohort study of patients diagnosed with unclassified bleeding disorder from 1998 to 2014.	'Patients with convincing symptoms of bleeding with no reproducible abnormality found with the standard tests of hemostasis'.<br><br>NOTE- term used in study is 'unclassified bleeding disorder (UBD)'	33 patients /78 procedures in total.<br><br>14 patients underwent dental/maxillofacial treatment.	13 patients underwent 19 dental extractions in total.<br><br>1 patient received a trigeminal nerve injection.	Oral TXA (ranging from 500mg to 1g 8 hourly commenced 24h before the procedure) and continued for 7-10 days.<br>Alternatively, a single dose of TXA (10mg kg^{-1}; maximum 1g) was administered intravenously prior to the intervention and continued orally (10-25 mg kg^{-1}; maximum 1g). TXA was also given in certain situations in combination with desmopressin (intravenous and subcutaneously) 0.3 µg kg^{-1}; maximum dose 28 µg.<br><br>Of the patients who received dental extractions, eight patients received TXA alone, one patient received DDAVP alone, and three patients received TXA and DDAVP.<br><br>One patient received no cover at all.<br><br>The patient who had a trigeminal nerve injection received TXA only.	In the patient who received no cover, there was bleeding 2 days post-extraction and an infected hematoma (note: this patient had a family history of bleeding issues).<br><br>One patient suffered an allergy to TXA and was later treated with desmopressin for a dental extraction.<br><br>A patient who had three dental extractions experienced prolonged bleeding following two extractions with just TXA, so the third extraction was completed with a combination of TXA and desmopressin, resulting in no further complications.<br><br>There was no bleeding associated with the trigeminal nerve injection.	• Retrospective data was used.<br>• Small patient numbers and the cohort of patients may not be a uniform group.<br>• No gold standard for the diagnosis of BDUC existed at the time of publication.<br>• Certain coagulation factor level tests were omitted, which may have identified other causes for the bleeding tendency.	Desmopressin (DDAVP) at 0.3 µg/kg (maximum 28 µg) and/or oral tranexamic acid (TXA) in patients with BDUC undergoing minor procedures is safe and effective.<br><br>The initial first-line treatment should be TXA. If bleeding persists, second-line treatment with desmopressin should be employed. If bleeding continues, platelets and/or fresh frozen plasma should be considered.<br><br>Etamsylate may be used as an alternative to TXA and in combination with DDAVP in patients with BDUC.
MacDonald et al, 2020[18]	Retrospective cohort study of registered patients with BDUC at a haemophilia centre from 1994 to 2018.<br><br>Mean age of presentation was 38.3 years.	Not explicitly defined in study.<br><br>NOTE- term used in study is 'unclassified bleeding disorder (UBD)'	124 patients /69 procedures	16 dental extractions	Patients were treated with either TXA at 15-25 mg/kg (up to 1 g) three times daily and/or desmopressin at 0.3 µg/kg subcutaneously or intravenously 30-60 minutes preprocedure, with fluid restriction for 24 hours afterward. Desmopressin was not considered for patients over 65 years of age or those with cardiovascular disease or hypertension.	Specific data for each dental extraction is not available. Bleeding during dental extraction was defined as 'abnormal bleeding noted by the patient or clinician'. One patient who was treated with TXA for dental extractions required DDAVP 12 hours later due to persistent bleeding, and another patient treated with TXA reported severe bleeding after a dental extraction.	• Previously registered patients with BDUC were not reviewed and potentially could have died from bleeding (data range from 1994 - 2018).<br>• Retrospective evaluation from a single centre.<br>• No standard definitions were used.<br>• Not all laboratory investigations were performed on all patients.	Haemostatic prophylaxis (TXA, desmopressin and platelets) is effective in patients with BDUC however clinical trials are required.
Furqan et al, 2020[19]	Retrospective review of adult patients (range 18-71 years) with von Willebrand's disease, platelet function disorder and BDUC who received desmopressin 0.15 ug/kg of bodyweight.	'Increased bleeding score with normal laboratory findings, despite extensive haemostasis testing'.<br><br>NOTE- term used in study is 'bleeding of unknown cause (BUC)'	17 patients/ 4 diagnosed with BDUC<br><br>33 procedures in total including 5 dental (15.2%), but specific procedures not revealed.	Not specified in study	Desmopressin (DDAVP) 0.15 µg/kg of bodyweight.	None reported (bleeding control was either excellent (87.9%) or good (12.1%).<br><br>However, hyponatremia and pre-syncope reported and attributed to DDAVP.	• Retrospective review of post-operative notes potentially resulting in reporting bias.<br>• Small patient group.<br>• Study only reports clinical efficacy in terms of bleeding control.<br>• Under representation of patients undergoing major surgeries.	Information is not very specific on patients who received dental treatment. General conclusion was half dose DDAVP is a safe and effective option in terms of bleeding control for patients undergoing minor surgical procedures.
Veen et al, 2021[17]	Retrospective analysis of BDUC patients greater than or equal to 12 years of age undergoing surgery.	'Clinically relevant bleeding history with no detection of haemostatic abnormalities after extensive laboratory investigation'<br><br>NOTE- term used in study is 'bleeding of unknown cause (BUC)'	53 patients/72 procedures.<br><br>7 patients underwent dental/maxillofacial surgery.	6 dental extractions and 1 jaw osteotomy	Patients who received dental/maxillofacial surgery were treated with TXA, desmopressin, or a combination of both. The exact doses and regimens are not revealed in the paper.<br><br>Two dental patients received no prophylactic treatment.<br><br>Of the patients who had dental extractions, 2 received no treatment, 2 received TXA, 1 received desmopressin, and 1 received a combination of TXA and desmopressin.<br><br>The patient who had an osteotomy of the jaw received TXA only.	Two patients who received dental extractions with TXA only had no issues.<br><br>One patient who had a dental extraction with desmopressin had no issues. Another patient who had a dental extraction with both TXA and desmopressin also had no issues.<br><br>The patient who underwent an extensive jaw osteotomy was covered with TXA only but later developed a major bleeding episode.<br><br>Of the two patients who had dental extractions with no prophylactic cover, one had no complications, while the other developed clinically relevant minor bleeding.	• Retrospective analysis<br>• Selection bias (tertiary care centre)<br>• Small sample size<br>• Information on pre- or peri-operative treatment and outcomes not always available	Patients with BDUC have a higher risk of bleeding compared to the general population, and this risk remains significant regardless of pre- or peri-operative haemostatic treatment.
Berkowitz et al, 2024[20]	Retrospective cohort study of adults with BDUC from 2015-2022.<br><br>Patients aged 18 years or above	'In comparison to ISTH ICC definition of BDUC, evaluation of Factor IX and XI was not explicitly required but patients needed to demonstrate normal aPTT and PT'	127 patients/129 procedures (76 minor and 53 major procedures)	Minor procedures included: simple dental extraction or dental procedures (exact procedure not revealed) in 5 patients.	No specific data is available. The majority of patients received either tranexamic acid or aminocaproic acid.	No bleeding issues reported with regards to dental/maxillofacial surgery.	• Periprocedural recommendations may not be representative of advice from all haematologists (setting was two academic medical centres).<br>• Non-whites are underrepresented.<br>• Exclusion of patients from the study who may have had BDUC (due to insufficient haemostatic workup).	Patients with BDUC have a low risk of haemostatic complications, provided that haemostatic prophylaxis is employed.

https://doi.org/10.1038/s41415-025-8815-z

	(mean age of 39.9 years).			Major procedures classified as: 3rd molar or complex extraction in 7 patients and 1 patient received a gingival graft.			• Exclusion of missing data may overestimate bleeding events.	

Abbreviations: TXA: Tranexamic acid
Desmopressin/(DDAVP): 1-deamino-8-D-arginine vasopressin
